# “And the little brain said to the big brain…” Editorial: Distributed networks: new outlooks on cerebellar function

**DOI:** 10.3389/fnsys.2015.00078

**Published:** 2015-05-15

**Authors:** Thomas C. Watson

**Affiliations:** ^1^School of Physiology and Pharmacology, University of BristolBristol, UK; ^2^Sorbonne Universites, UPMC Univ Paris 06, Neuroscience Paris Seine, UMR CNRS 8246, INSERM 1130, Institut de Biologie Paris Seine, Cerebellum Navigation and Memory TeamParis, France

**Keywords:** cerebellum, networks, purkinje cell, autism spectrum disorders, internal model, navigation, oscillations, cerebellar nuclei

The purpose of this research topic is to showcase recent anatomical, physiological, and clinical studies revealing how the cerebellum is embedded within distributed neural networks recruited during both direct motor functions plus cognitive and other domains. The topic comprises a total of 15 articles that together span experimental, theoretical, methodological approaches, and reviews of the literature.

In recent decades, converging lines of evidence indicate that the roles of the cerebellum extend beyond direct motor control to include interactions with higher centers associated with cognition. Identifying the functional roles of the cerebellum within these wider brain networks is therefore of critical importance when developing methods to alleviate symptoms of both motor and non-motor disorders (as recently exemplified by Chen et al., 2014 in a mouse model of rapid onset dystonia).

The papers in this research topic highlight several themes relevant to deciphering the cerebellar position within the wider brain-web. Voogd (2014), through his detailed review, emphasizes the importance of understanding structure to help understand function. Indeed, he describes numerous ascending pathways linking discrete cerebellar zones/modules to cerebral regions. The article also serves as a reminder that, despite best efforts, we still understand cerebellar systems rather superficially.

Anatomical studies are fundamental to shedding light on this problem and Ruigrok and Teune (2014) demonstrate how the use of intricate retrograde neural tracing techniques can help chart the route of the all-important “modular” cerebellar output via the deep cerebellar nuclei. They identify two ascending cerebellar pathways in which cerebellar output to premotor areas such as the red nucleus can supply simultaneous feedback to both the mossy and climbing fiber systems, and can act in concert with a designated GABAergic nucleo-olivary circuit. How these findings extend to cerebellar connectivity with non-motor brain regions will be of great interest and ultimately, discrete manipulations of these specific pathways will allow us to understand their importance in controlling cerebellar processes. Watson et al. (2014) employed electrophysiological techniques to map out physiological interactions between the cerebellar fastigial nuclei and the prelimbic cortex in anaesthetized and awake rats. This study demonstrates clear physiological coupling between the structures: local field potential (LFP) oscillations in the theta frequency range (5–10 Hz) in both the prefrontal cortex and cerebellar fastigius nucleus are synchronized according to behavioral demands. Along similar lines, Frederick et al. (2014) have demonstrated the presence of a cerebello-striatal interaction linked to circadian rhythms and dopamine levels. Like Watson et al. (2014) they have also used coherence analysis of LFPs but in this case recorded in dorsal striatum and Crus 1 of the cerebellar hemisphere. The authors reveal correlated low (0–3 Hz) and theta (3–8 Hz) frequency rhythmic oscillations within both regions in anaesthetized rats that vary depending upon time of day and dopamine levels. Together, these two papers highlight the utility of LFP recording in rodent models in understanding distributed networks that involve the cerebellum.

In keeping with these results, Parker et al. (2014) have suggested an experimental methodology to test the importance of cingulate-cerebellar connectivity in rat models of schizophrenia. By combining optogenetic and anatomical tracing techniques they suggest a strategy to selectively modulate cerebello-cingulate pathways in an attempt to alleviate abnormal behavior. The outcomes of future rodent studies adopting this approach will prove especially valuable when viewed in a translational context alongside human neuroimaging and clinical studies.

In this respect, the review paper of Reeber et al. (2013) usefully summarizes the multitude of non-motor human disorders associated with disrupted cerebellar function and outlines their genetic, electrophysiological, and behavioral bases. Amongst others, they highlight a recent landmark study in which the loss of the Tsc1 gene from mouse cerebellar Purkinje cells (Tsai et al., 2012) is sufficient to cause social impairments, cognitive defects and abnormal vocalizations –core features of autism spectrum disorder (ASD). These behaviors were successfully blocked in mutants treated with the mTOR inhibitor rapamycin. Such rodent models will be essential to understanding cerebellar circuit function in disorders that encompass symptoms beyond direct motor abnormalities, particularly developmental disorders that may compromise the strict patterns of cerebellar connectivity laid down during development. Furthermore, as Stoodley (2014) has demonstrated in this research topic, fMRI experiments in humans identify that clearly localized disruption of distinct cerebellar anatomical clusters, and the networks they connect to, exists in disorders such as ASD, dyslexia, and ADHD. The importance of appropriately controlled analysis of fMRI data is also described here by Michael et al. (2014). Alongside the contribution of Ordek et al. (2014), who describe the effect of percussive damage to cerebellar cortical processing of sensory information, these studies collectively emphasize the vulnerability of the cerebellar cortex and the diverse symptoms its dysfunction can trigger.

The organization of the cerebellum is often described as a uniform circuit, but this belies the complexity of its rich molecular architecture. Our understanding of the functional significance of these expression patterns is still in its infancy. One of the best known examples is the parasagittal striping that results from the non-uniform expression of zebrin II/aldolase C in Purkinje cells. Hawkes (2014) provides a review of the importance of this molecular architecture in long term depression (LTD) at the parallel fiber- Purkinje cell synapse. But many questions remain unresolved, including how the presence of specific molecular markers within select Purkinje cell populations might alter their firing patterns in response to afferent input.

Within this review topic this important question is beginning to be addressed. Physiological evidence suggests that Purkinje cell firing patterns are not homogenous across the cerebellar cortex (De Gruijl et al., 2014; Xiao et al., 2014) and the control of cerebellar population firing is indeed influenced by factors such as zebrin expression (Tsutsumi et al., 2015). Additionally, the pattern of complex spike waveform seen within Purkinje cells is modulated by the synchrony of firing within the inferior olive (Lang et al., 2014), which provides details of how afferent input, carried via olivo-cerebellar pathways, can influence cerebellar spiking. Whether this synchrony level is selectively gated or modified by olivary inputs remains to be seen, but this finding provides a potential route via which the activity in populations of Purkinje cells could vary depending upon upstream activity in non-cerebellar regions (see also Ros et al., 2009), and also possess the capacity to process motor and non-motor information in a differential manner. Future studies utilizing optogenetic manipulations restricted by zebrin markers should provide a method to probe the functional significance of these neuronal populations (e.g., Tsubota et al., 2013).

At a behavioral level, our understanding of cerebellar contributions remains far from complete. In their review, Rondi-Reig et al. (2014) describe the anatomical pathways through which the cerebellum may connect to known navigation centers (e.g., hippocampus) and highlight the contribution of cerebellar monitoring of sensory information to maintaining body position in space. They extend the work of Dean et al. (2010) to suggest that during navigation, the cerebellum may filter out predictable sensory signals and preferentially convey novel sensory information to hippocampal circuits to facilitate the maintenance of cognitive maps (as manifest in the activity of CA1 place cells). Such a cerebello-hippocampal network model may indeed rely upon the predictive nature of cerebellar processing. In relation to internal models, Popa et al. (2014) describe how Purkinje cell simple spike firing can accurately predict movements across a variable timescale while also detecting errors within the motor domain. They suggest that this capacity may extend to support associative learning, sequencing, working memory, and forward internal models in non-motor domains. Indeed, the ability to predict both the motor and non-motor consequence of movements is likely to be fundamental to the generation and execution of appropriate goal-directed behaviors. Cheron et al. (2014) build upon preliminary data from an Angelman syndrome mouse model to suggest that normal synaptic plasticity processes (specifically, LTD) in the cerebellar cortex modulate inhibition/excitation balance within cerebral cortical areas, thereby influencing plasticity in disparate regions such as the barrel cortex. By using transcranial cerebello-cerebral direct-current stimulation to relieve symptoms of spino-cerebellar ataxias, Grimaldi et al. (2014) have also demonstrated the importance of cerebro-cerebellar dialog in generating appropriate muscle control in upper limbs.

The wide-ranging contributions made to this research topic reinforce the view that the cerebellum plays a role in a variety of processes that are fundamental to the generation of appropriate behaviors. While we know a great deal about the intrinsic anatomy of the cerebellum, it is also clear that we are still some way from gaining a full understanding of the functional significance of this intricate organization. Meanwhile, from a clinical perspective, much can be gained by further investigating cerebellar contributions to a variety of neuropsychiatric disorders. We are entering an exciting period in cerebellar research—the adoption of behavioral, genetic, and translational approaches (as often employed when deciphering other “big-brain” circuits) will surely lead to important advances in our understanding of cerebellar roles in health and disease.

## Conflict of interest statement

The author declares that the research was conducted in the absence of any commercial or financial relationships that could be construed as a potential conflict of interest.
